# Goose Embryonic Development, Glucose and Thyroid Hormone Concentrations, and Eggshell Features Depend on Female Age and Laying Period

**DOI:** 10.3390/ani12192614

**Published:** 2022-09-29

**Authors:** Joanna Kucharska-Gaca, Marek Adamski, Jakub Biesek

**Affiliations:** Department of Animal Breeding and Nutrition, Faculty of Animal Breeding and Biology, Bydgoszcz University of Science and Technology, Mazowiecka 28, 85-084 Bydgoszcz, Poland

**Keywords:** age, blood biochemistry, embryo, goose, hatching egg, incubation, reproductive flock, thyroxine, triiodothyronine

## Abstract

**Simple Summary:**

Embryonic development is a crucial issue in the breeding and production of geese. Since geese are kept for four seasons of reproduction, the age of females may affect the development of geese. During incubation, many processes take place inside the egg. With development, organs begin to form, and thyroid hormones and glucose begin to play an essential role in the regulation of these processes. These processes are related to the incubatory conditions and the temperature on the surface of the eggshell. Thus, the eggshell’s physical characteristics can influence a goose’s embryonic development. It was shown that geese’s age (1- to 4-year-old) and laying period (the beginning, the peak, and the end) affect the embryonic development of geese. The concentration of triiodothyronine and thyroxine and the eggshell’s characteristics, including thickness, density, and temperature, were also affected by those factors. This knowledge can support the production of goslings and hatching technology.

**Abstract:**

This study aimed to evaluate embryonic development; analyze the glucose, triiodothyronine (T3), and thyroxine (T4) concentrations in the blood of embryos and goslings; and assess the structure and temperature (EST) of the eggshell. The eggs that were analyzed were from four laying seasons of White Kołuda^®^ geese at three periods (90 eggs × 4 groups × 3 periods). The different embryo proportions, fetal membranes in the egg, and sizes of internal organs indicate a different growth rate and degree of embryo development depending on the laying age and laying period. The goose age influenced the hormone concentrations in the embryos’ blood on the 28th day of incubation, which supports a relationship between the females’ age and development. The eggshell thickness and density change depending on the laying age and the laying period. A decrease in eggshell thickness in the eggs up to the third season was found after the 16th day of incubation (simultaneously, the density showed an increasing trend). A lower EST distinguished the eggs from the oldest geese in the first half of the hatch. The formation of the chorioallantois membrane was associated with an increase in EST in the oldest geese.

## 1. Introduction

During the growth and maturation of female oocytes, thyroid hormones, e.g., thyroxine (T4) and triiodothyronine (T3), are stored in the yolk sac’s reserve material [1]. According to Christensen et al. [2], the age of laying hens influences the number of deposited thyroid hormone substrates. The hormone quantity deposited by chickens may depend on their age. In the embryonic tissues, there may be a different amount of the deiodinase enzyme, which determines the conversion of T4 to T3. These hormones regulate embryonic development. They play a role in tissue structure, growth, and differentiation. Growth mainly involves cell proliferation but may be associated with an increased cell size. It affects the process of bone formation and the growth and development of the heart and liver of embryos [3,4]. Thyroid hormones may indirectly influence the building of muscle tissue. This process can be affected by the expression of specific forms of actin and isoforms of myosin, as confirmed in the study by Gardahaut et al. [5]. According to Hughes et al. [6], thyroid hormones influence the breakdown of fast twitch and slow twitch fibers in muscles through changes in the mRNA expression of the *MyoD* gene and myogenin. T3 and growth hormone (GH) are essential components for fully expressing tissue growth and development.

The release of these hormones increases during the incubation and is highest in the hatching chicks and just after hatching, indicating the interaction of hormones. Stockdale [7] found that there are intense processes of fetal myoblast differentiation and mature myoblast proliferation (satellite cells) that determine the final size of the skeletal muscles of the birds. It can be presumed that an increase in the thyroid hormone concentration after two weeks of incubation may affect the increase in muscle mass [8]. Removing the thyroid gland in chickens caused a significant reduction in growth rate, and the negative effect could only be normalized by administering T3 or T4 [9]. Hormones, including T3 and T4, can regulate the process of myogenesis. The influence of the female turkeys age on the concentration of the hormones T3 and T4 has also been studied [2].

Glucose is an essential nutrient during embryogenesis. Carbonates are especially important in the late incubation phase as they are responsible for meeting the metabolic needs of the embryo. The embryos prefer glucose over fatty acids for energy production [10]. The availability of glucose in the last days of incubation may depend on the origin, age of the laying, or storage time of the eggs [11].

Previously, it was shown that light and temperature determine the hatching results of birds [12,13] and slaughter parameters [14,15]. The relationship between the age of the female and the development of the offspring’s muscles was not analyzed. The embryonic temperature depends on the air temperature, the yolk size, and the lipid content. It may be influenced by the age of the female or the egg-laying period. Powell et al. [16] noticed that chicks that hatched earliest (in the same incubation period) had better muscle development in the first week of life.

It should be noted that the incubation length depends on many factors, including the age of the laying hen and the egg’s weight. Thus, the temperature can also determine the embryonic development of birds. It is also related to the physical properties of the eggshell, including the thickness and water loss from the egg during incubation [17].

Considering the above, it seems justified to research the course of the embryonic development of geese, while considering the influence of the age of females. This study aimed to assess the embryonic development of geese; analyze the concentration of glucose, triiodothyronine (T3), and thyroxine (T4) in the blood of embryos and goslings from differently aged female flocks; and assess changes in egg weight and eggshell thickness, density, and temperature during incubation.

## 2. Materials and Methods

The research was carried out with the consent of the Local Ethical Committee in Bydgoszcz (no. 30/2015).

### 2.1. Goose Farm and Egg Collection

Hatching eggs were obtained from four breeding flocks of White Kołuda^®^ geese kept on a farm in the Kuyavian-Pomeranian Voivodeship in Poland. The females were in the 1st, 2nd, 3rd, and 4th seasons of reproduction. The flocks numbered 1500 geese. The male-to-female ratio was 1:4/5. Ganders were the same age as the geese. All breeders were kept the same way (buildings with access to the range). The light cycle was consistent with natural light (January to June). During the period of reproduction, the lighting was standard: 10 h of light and 14 h of darkness. The building was not equipped with an additional source of heating. The nutrition was balanced according to the standards of the parent goose flocks (approx. 11.21 MJ of metabolizable energy and 14.40% of crude protein in 1 kg of feed). Hatching eggs for the study were collected at the 4th week of laying (start), 13th week (peak), and 20th week (end). The laying cycle lasted from January to June. The eggs were chosen randomly. Eggs of irregular shape or that were too small/large were eliminated. A total of 1080 hatching eggs were assessed. For embryonic development and sampling analysis, 90 eggs were obtained from each breeding flock at three laying periods. The eggs were collected three days before the planned incubation.

### 2.2. Egg Incubation and Sample Collection

Hatching eggs were disinfected with Vircon and stored at 10 °C and 70–75% relative humidity. Before incubation, the eggs were weighed on a PS 750/X scale (Radwag, Radom, Poland) and again disinfected. The eggs were marked with a group symbol and an ordinal number. Hatching was carried out in a one-shot incubator (Jarson, Gostyń, Poland). The air temperature in the hatching chamber (1–26 days) was 37.7 °C, and the relative humidity was 55%. On the 2nd day of hatching, the cooling of the chamber began, which consisted of opening the apparatus for 20 min twice a day. The treatment was repeated until the eggs were transferred to the hatcher. Starting on the 9th day, the airing and sprinkling procedure of the eggs outside the chamber was carried out. Starting on the 16th day of incubation, the eggs were manually rotated 180° along the egg’s long axis. Rotation and airing were performed once a day. A temperature of 37.4 °C and a humidity of 75% were set in the hatcher chamber (27–31 days). The hatcher chamber was aired once a day for 20 min. On day 31, the goslings hatched. The degree of embryo development in individual experimental groups was assessed at three incubation dates: the 16th, 22nd, and 28th day. Fifteen eggs of each age group (at each date) were randomly selected.

### 2.3. Glucose, T3, and T4 Hormones Concentration

Blood was collected from the chorioallantoic membrane before assessing the inside of the egg. The material was collected from the blood vessels of the chorioallantoic membrane using a 0.2 mm wide needle. Before blood sampling, each egg was illuminated with an egg X-ray machine (to determine the exact location of the blood vessels). An eggshell fragment (an area of approx. 1 cm^2^) was cut out. A dental drill was used (Velleman, Gavere, Belgium). A cut piece of eggshell was removed without damaging the sub-shell membranes of the egg. Blood was drawn into test tubes through a drilled hole.

The blood was centrifuged (analysis conditions: 10 min, 4000 rpm). Glucose, triiodothyronine (T3), and thyroxine (T4) were determined in the plasma. The glucose content in the blood plasma was determined by spectrophotometric method, using the Epoll-20 biochemical analyzer at a wavelength of 520 nm with the use of ready-made reagent kits (BioMaxima, Lublin, Poland). T4 and T3 hormones were determined by radioimmunoassay using RIA-T4-J125 and RIA-T3-J125 ready-made kits (DIAsource ImmunoAssays, Ottignies, Belgium). The activity of the sediments was measured in an automatic gamma radiation counter.

After hatching, 8 one-day-old goslings were randomly selected from each group. Blood was also taken by decapitation of the head. The blood was centrifuged, and the plasma was designated for determining glucose, T3, and T4.

### 2.4. Embryo Assessment

The eggs were cut open, and the weights (g) of the embryo, yolk sac, chorioallantoic membrane (CAM), heart, and liver were determined (weight PS 750/X, Radwag, Radom, Poland). The embryo proportion in the egg and the share of the embryo’s organs were calculated. The following formulas were established:

- embryo (%)$=\frac{\mathrm{embryo}\;\mathrm{weight}\;\left(g\right)}{\mathrm{egg}\;\mathrm{weight}\;\left(g\right)}\times 100$,
- CAM (%)$=\frac{\mathrm{CAM}\;\mathrm{weight}\;\left(g\right)}{\mathrm{egg}\;\mathrm{weight}\;\left(g\right)}\times 100$,
- yolk sack (%)$=\frac{\mathrm{yolk}\;\mathrm{sac}\;\mathrm{weight}\;\left(g\right)}{\mathrm{embryo}\;\mathrm{weight}\;\left(g\right)}\times 100$,
- heart (%)$=\frac{\mathrm{heart}\;\mathrm{weight}\;\left(g\right)}{\mathrm{embryo}\;\mathrm{weight}\;\left(g\right)}\times 100$,
- liver (%)$=\frac{\mathrm{liver}\;\mathrm{weight}\left(g\right)}{\mathrm{embryo}\;\mathrm{weight}\;\left(g\right)}\times 100$.

The embryo length (cm) from the beak to the middle toe was measured with tape and graph paper (with an accuracy of 0.1 mm).

The eggshells were collected, and the density and thickness were determined [18]. The eggshell surface temperature (EST) was monitored using a non-contact thermometer with a laser tip. Measurements were made on the eggshells’ surfaces placed at different incubator levels at the same points throughout the incubation period.

After hatching, the goslings were weighed, and their share in the egg was calculated.

### 2.5. Statistics

The data were processed in Statistica 12.5 PL program (Statsoft TIBCO Software Inc., Cracow, Poland). The mean values and standard deviation (±SD) were calculated. An analysis of variance was used to determine individual differences in the calculations (effect of parent flock age and laying period of geese). The data were checked for homogeneity of variances (Levene’s test) and the normality of data distribution (Kolmogorov-Smirnov test). HSD Tukey’s test was used, with *p*-value < 0.05. Interaction between parent flock age and laying period was also analyzed (*p*-value < 0.05).

## 3. Results

### 3.1. Glucose and T3 and T4 Hormone Concentration

#### 3.1.1. Glucose

The age of the laying geese was not found to influence the glucose concentration in the plasma of goslings during embryogenesis (*p* > 0.05). Therefore, the data are presented in the form of a description. On day 16, the glucose concentration in the tested samples was 128.8 ± 13.7 mg/dL (group I), 123.7 ± 23.1 mg/dL (group II), 129.1 ± 18.1 mg/dL (group III), and 117.1 ± 34.3 mg/dL (group IV). On day 22, the levels of blood glucose increased in comparison with day 16: 195.3 ± 40.5 mg/dL (I), 192.2 ± 36.3 mg/dL (II), 189.3 ± 54.4 mg/dL (III), and 182.2 ± 30.9 mg/dL (IV). Plasma glucose concentration showed a downward trend on day 28: 178.2 ± 18.8 mg/dL (I), 167.1 ± 28.8 mg/dL (II), and 178.9 ± 45.0 mg/dL (III), except for group IV—185.8 ± 31.4 mg/dL. Its highest concentration was recorded after hatching, and the mean values were as follows: II—191.2 ± 12.9 mg/dL < IV—208.9 ± 32.4 mg/dL < I—212.5 ± 19.2 mg/dL < III—222.1 ± 31.4 mg/dL.

#### 3.1.2. Triiodothyronine (T3) and Thyroxine (T4)

**Figure 1:**
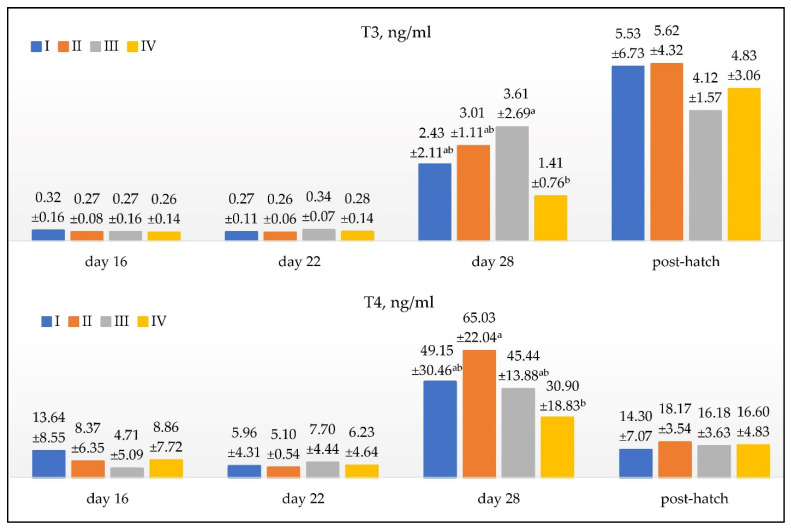
The concentration of triiodothyronine (T3) and thyroxine (T4) in the blood serum of embryos and goose chicks depends on the age of the parent flock. Values are the mean ± SD (standard deviation) ^a,b^; mean values marked with different letters differ significantly; *p* < 0.05; I, 1-year-old geese; II, 2-year-old geese; III, 3-year-old geese; IV, 4-year-old geese; n = 15/per group; n = 8/per group post-hatch.

Figure 1 shows the concentration of triiodothyronine (T3) and thyroxine (T4) in the blood serum of the embryos and goslings, depending on the females’ age of the parent flock.

It was shown that by the 16th and 22nd day of incubation, the concentrations of T3 in the plasma were similar. In the following days of hatching, there was an intense increase in hormone concentration. The results showed that the age of the parent flock differentiates goose embryos in terms of their plasma T3 concentration on the 28th day of hatching (*p* < 0.05). The highest concentration of the analyzed hormone was recorded in group III and the lowest in group IV. In the embryos from groups I and II, the plasma concentrations of T3 were similar. On the first day of the goslings’ life, the hormone concentration was high: 4.12–5.62.

The females’ age significantly affected the concentration of T4 in the plasma of the embryos only on the 28th day of hatching (*p* < 0.05). The highest values were recorded in group II and the lowest in group IV. In the remaining groups, the hormone concentration was similar. On the 16th day of incubation, the T4 concentration for all the groups was not different (*p* > 0.05). On day 22, the values were also similar. The hormone concentration in the goslings’ blood plasma in the age groups was 14.3–8.17 ng/mL.

### 3.2. Embryo Assessment

For all measurement dates carried out during incubation, the hatching eggs differed in weight (*p* < 0.05, Table 1, Table 2 and Table 3). On the 16th, 22nd, and 28th day of hatching, the weight of the embryos from group I was the lowest compared to the embryos from group IV. The difference in the embryos’ weight between the youngest and the oldest females increased during the incubation. Regardless of the incubation date, the highest weight of the embryos was found in group IV. The embryos obtained from eggs at the beginning of laying were characterized by the highest body weight (*p* < 0.001) compared to the other periods on day 22 (Table 2) and day 28 (Table 3).

On the 16th day of incubation, the highest embryo proportion in the egg weight group was recorded in group I (Table 1). The same was true of the following laying period. On day 28, the lowest share of the embryos in the egg was found in group IV (*p* = 0.011, Table 3).

In different laying periods, it was found that the highest share of embryos was found at the peak and the end of laying (*p* < 0.001, Table 1).

Measurements of the embryos’ length showed that the embryos from groups II and III were the longest until the 22nd day of incubation. At these evaluation dates, the shortest embryo was recorded for group I. On day 28, the embryos from group I were shorter than in the other groups. Until day 22, the embryos in the eggs from the beginning of laying had the shortest body length. On the 28th day, it was found that the embryos from this period were longer than the other groups (*p* < 0.001, Table 1, Table 2 and Table 3).

On the 16th day of hatching the eggs from groups III and IV, the share of the chorioallantoic membrane (CAM) was significantly higher than in groups I and II. On the 28th day of incubation, the highest proportion of membrane was found in the eggs of group IV among all the other groups (*p* < 0.001). The laying period affected only two measurement dates: the 16th and 22nd day of hatching. Eggs from the beginning period were characterized by a higher proportion of CAM than at the end (Table 1, Table 2 and Table 3).

The share of the yolk sac was assessed on days 22 and 28. On the 22nd day of incubation, the eggs from groups II and IV were characterized by the highest proportion of yolk sac. In the final stage of incubation, it was shown that the yolk sac proportion in the egg increased with the females’ age (*p* < 0.001). Regardless of the laying period, the share of the yolk sacs during embryogenesis was highest in the eggs obtained at the peak of laying (Table 2 and Table 3).

On the 16th day of hatching, the highest share of hearts was found in group II (*p* = 0.028). On day 28, the highest share of hearts was found in groups II and III (*p* = 0.024). Following the impact of the laying period, it was found that up to the 22nd day, the highest share of hearts was characteristic of eggs obtained at the peak of laying (*p* < 0.001). The highest percentage of the embryonic hearts on day 28 was found in eggs from the end of laying (*p* = 0.032) (Table 1, Table 2 and Table 3).

On the 16th day, the highest percentage of livers was found in the embryos from group II and the lowest in group I (*p* < 0.001, Table 1). An influence of the laying period on the embryonic liver share was noted on the 16th and 28th days. On day 16, a higher liver share was found in the eggs obtained at the beginning and peak of laying (*p* = 0.001, Table 1). On day 28, the opposite trend was demonstrated. As the laying period progressed, the embryos’ liver shares increased (*p* = 0.004, Table 3).

An interaction of the females’ age and laying period was demonstrated for the egg weight, embryo length, and percentage of CAM in the egg and the heart and liver in the embryo on the 16th day (*p* < 0.05, Table 1). On day 22, an interaction between the females’ age and the laying period was noted for the embryos’ length and the yolk sac content in the egg (*p* < 0.05, Table 2). On day 28, an interaction between the factors influencing the embryo length was demonstrated (*p* < 0.001, Table 3).

In each of the females’ age groups and laying periods, the thickness of the eggshell of the hatching eggs decreased with successive days of incubation (*p* < 0.05).

The thickest eggshells were found in eggs obtained at the beginning of laying. With each subsequent laying period, the thickness of the eggshell decreased (*p* < 0.001). However, a significant decline in the eggshell thickness in different laying periods occurred at different hatching stages. On days 16 and 22, a higher thickness was noted at the beginning and the peak of the laying period. At the end of the laying period, a decrease in eggshell thickness was noticed on day 31 (Table 4).

The females’ age influenced the eggshell density (Table 4). A higher density of the eggshells was found in group III compared to groups I and II (*p* = 0.005) on day 22. On the 31st day, the eggshell density was higher in group II than in the others (*p* < 0.001). In addition, on day 31, a lower eggshell density at the peak of the laying period was found (*p* < 0.001). Comparing the incubation days, for group I, the density was lower on the 16th day (*p* < 0.001). In groups I and II, the density increased with the number of days (*p* < 0.001). At the end of the laying period, a higher eggshell density was found on the 31st day of incubation (*p* < 0.001).

An interaction between the main factors was found only in the density on day 31, after hatching (*p* < 0.001).

An influence of the females’ age on water losses was noted on the 4th day of the hatching. The highest loss of water was recorded in groups II, III, and IV (*p* < 0.001). The analysis showed that a different weight loss characterized the eggs during the incubation depending on the laying period. The lowest degree of water evaporation was found at the beginning of laying, compared to the peak and the end. Throughout the incubation, different laying periods were characterized by various water loss values (*p* < 0.05). The females’ age and laying periods had a different effect on the degree of water loss during incubation. The interaction between the factors on the value of this feature was found on the 4th day of incubation (Table 5).

**Figure 2:**
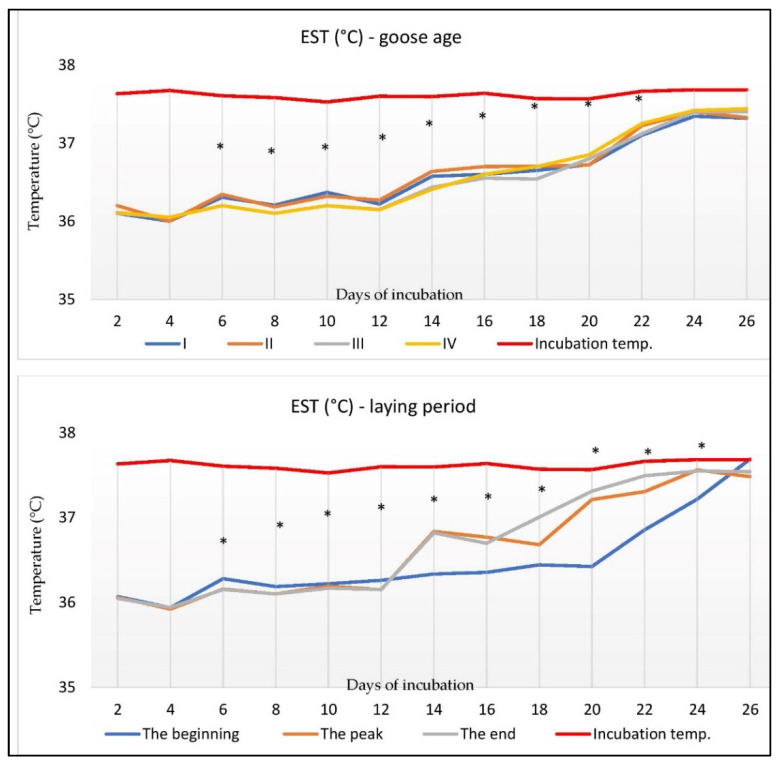
The eggshell surface temperature (EST) of goose eggs during incubation depends on the age of the females and the laying period. *, mean values differ significantly (*p* < 0.05); I, 1-year-old geese; II, 2-year-old geese; III, 3-year-old geese; IV, 4-year-old geese; n = 15/per group.

The eggshell surface temperature (EST) of the goose eggs during incubation depended on the age of the females and the laying period, and it is presented in Figure 2. Differences between the age groups were noticed from the 6th to 22nd days of incubation (*p* < 0.05). In the first two days of hatching, the EST of the eggs ranged from 36.1 to 36.2 °C. Until the 16th day of hatching, a higher EST was shown in the eggs from groups I and II compared to groups III and IV. After the 16th day, higher values in groups III and IV were found compared to groups I and II. The highest EST was recorded on the 24th and 26th days.

The laying period significantly influenced the changes in the EST between the 6th and 24th day of incubation. On the 4th day of incubation, a decrease in the EST was noted. Until day 20, the eggs at the beginning of laying were distinguished by a slow increase in EST, followed by a rapid increase. At the beginning of the laying, the EST was higher than on the other dates (up to the 12th incubation day). It was noted that the EST increased intensively from the peak and end of laying (*p* < 0.05).

The weight and percentage of goslings, which are dependent on the females’ age and laying period, are described here. The highest body weight of the goslings was present in group IV at 142.3 g, and the lowest in group I at 101.2 g. In group II, the weight was 114.5 g, and in group III it was 129.4 g (±SD: 3.20–8.10). Significant differences between all of the female age groups were found (*p* < 0.05). The percentages of goslings in the eggs were in the following order: II—66.1 < I—66.5 < III—67.4 < IV—67.7% (±SD: 0.24–0.56). Higher shares of goslings were found in groups III and IV compared to groups I and II (*p* < 0.05). The goslings’ weights by the laying period were 127.3 g (the beginning), 119.9 g (the peak), and 115.7 g (the end). Significant differences were found between each laying period (*p* < 0.05). A higher share (*p* < 0.05) of goslings in the eggs was recorded at the beginning and peak of the laying period (67.2% ± 0.23 and 67.3% ± 0.47, respectively) compared to the end of the laying period (66.3% ± 0.52).

## 4. Discussion

Rząsa et al. [19] determined the effect of the genotype (two strains, W33 and W11, of white Italian goose) on the embryo’s thyroid hormone concentration. The T4 hormone concentration increased gradually from the 15th day of embryogenesis and then decreased on day 29. A dynamical increase in the T3 concentration from day 15 to hatching was also reported. Our results were similar to those of the cited authors. An increase in the T3 hormone concentration occurred during the hatching period. This may be related to the thermogenic reaction at the end of incubation [19]. Reyns et al. [20] and Lu et al. [21] showed that the increase in hormones in the blood plasma of embryos is associated with the breakthrough of the egg’s air chamber and the change from chorionic to pulmonary respiration. This indicates the significant role of thyroid hormones in lung maturation and in triggering the pulmonary respiration reflex [22].

The influence of age on all the analyzed features was demonstrated in research with turkey embryos and their hormone concentrations [2]. The authors found a higher concentration in the younger layers group than in the older ones on the 24th day of incubation. The influence of age on the T3 concentration was shown on the 27th day of hatching, i.e., in the external hatching phase. Higher thyroid hormone levels may be directly related to the hatching process. The hormones support the growth and development of the muscular system, also known as the ‘hatching muscle’. Its primary function is to support the embryo in piercing the membranes and shells during hatching. The hatching muscle in chickens fills up with interstitial fluid and becomes larger. After hatching, it becomes smaller [22]. It can be suggested that during the inner hatching, the higher proportion of the thyroid hormone in groups I, II, and III prepares the smaller embryos to break the thicker eggshell.

On the 28th day of hatching, the egg’s temperature might also influence a reduction in T3 concentration in the plasma of goose embryos from 4-year-old birds [23]. A high egg temperature causes a decrease in the T3 concentration in the embryo’s plasma. This phenomenon explains the goslings’ lower body temperature. Pietras et al. [24] observed the influence of a high temperature and relative air humidity while assessing the effect of thermal stress during bird rearing on the thyroid hormone concentration. The thyrotropic axis activity of the birds was reduced, with a decreased thyroid hormone concentration [24]. Moreover, a low concentration of thyroid hormones may extend the hatching time and delay hatching [25].

Our research found an effect of the females’ age on the embryos’ weight during incubation. Wilson [26] found that the females’ age influenced the chick’s weight only in the second half of the incubation period. In hens, the effect was recorded from the 11th day of hatching. This phenomenon may be related to the rapid transfer of lipids from the yolk to the embryo. In the study of Nangsuay et al. [27], the influence of the females’ age on the body weight of chicken embryos from the 14th day of incubation was confirmed.

The percentage of an embryo in the egg was higher in the group of young geese compared to the older layers. These differences persisted until the 28th day of hatching. However, after hatching, a lower share of embryos in the eggs of the laying geese occurred in groups I and II, while a higher share was recorded for groups III and IV. The yolk sac retraction in geese occurs on the 29th day of hatching [28]. The size of the yolk sac could influence the increase in the proportion of the embryo in the egg. This phenomenon is associated with the resorption of the larger yolk and the larger mucosa of the yolk sac and the vascular system [29]. In our research, the laying period influenced the share of the embryos in the egg in the first half of the hatching period (differences were noted only on the 16th day of incubation). Yadgary et al. [30] found that the proportion of embryos in the egg was higher in younger chickens (30 weeks) than in older hens (50 weeks). It can be assumed that the embryonic development in heavier eggs obtained at the beginning of laying is slower in the first two weeks of hatching.

It was found that the laying period differentiated the geese in terms of body length. Our results are similar to those of Şahan et al. [29], while the body length of the offspring from the older layers was higher than that of the younger ones.

The higher weight of the goslings of the older geese eggs was not proportional to the higher weight of the internal organs. The liver share in the goose embryos of different females’ age groups changed during hatching. According to Morita et al. [31], the liver may be related to the physical characteristics of the eggshell, which indirectly determines the rate of metabolism and gas exchange. The authors found larger embryonic livers from eggs with a thinner eggshell, a higher conductivity, and several pores. In our research, the laying period influenced the size of the liver. Analyzing embryonic development at four laying periods (females aged: 29, 41, 53, and 65 weeks), Hudson et al. [32] found that on the 21st day of incubation, the percentage of the liver was higher in egg embryos from younger hens compared to older hens. The differences between the age groups decreased with successive days of incubation, and no differences were found on the 21st day of hatching [2].

The heart share in all the analyzed periods was the highest in groups II and III and at the end of the laying period. Embryos from the beginning of laying were characterized by a higher heart share up to the 22nd day. Morita et al. [31] obtained similar results by comparing two laying periods (the 29th and 60th week of life). The authors suggested that most features increased proportionally with the embryo’s development (embryo’s weight and length and liver weight) or decreased (fetal membranes). However, the opposite trend was shown in the case of the heart’s involvement in the embryo. Regardless of the females’ age or the laying period, the heart development was more intense in the first half of incubation. These results are similar to those of Zhang and Burggren [33]. The increase in the heart weight during embryonic development of the hens was disproportionately higher than that of the embryo in the first 10 days of incubation. However, the development and growth of the heart were slower later on. 

The fetal membrane growth determines the embryo’s proper development [34]. In hens, the chorionic allantoic (CAM) membrane begins to form on the 4th day of incubation and develops by the 12th day. A germinal membrane serves as a gas exchange surface, and a dense capillary network supports its function. Mortola and Awam [35] found that the CAM weight on day 17 of incubation was higher in L-size eggs (>63 g). The pattern of CAM growth during incubation was similar in different weight groups of eggs. By controlling the allantoic development in goose eggs, it was found that the change of the goose eggs’ weight and circumference during the laying period is related to the growth rate of the allantoic membrane (TWO) [34]. A higher TWO index characterized the eggs obtained at the end of laying. Our research found an influence of the females’ age and laying period on the CAM weight. However, the relationship between the CAM size and egg weight was not excluded, as the age groups and the individual laying periods differed in egg weight. The differences in the membrane proportion in the first half of the incubation were due to the females’ age. At the end of the incubation period, the CAM share was the highest in group IV. The effect of the laying period was noticed up to the 22nd day of incubation. The development of the CAM, just below the intimal surface, ensures an adequate degree of albumen absorption, influencing the growth of the embryo and its ability to hatch [36].

The yolk sac’s nutrient content directly depends on the weight of the hatching eggs and indirectly on the breed and females’ age. The embryo uses the yolk components during incubation and the first days of life [37,38]. Our research confirmed the influence of a goose’s age and laying period on the share of the yolk sac in the egg. According to Yadgary et al. [30], the size of the yolk sac is related to the weight of the yolk. The higher yolk weights of the eggs of meat-type hens at the end of the laying period increased the weight of the yolk sac. It was shown that the yolk sac’s weight throughout the hatching period was higher in the eggs obtained at the 50th week of laying than at the 30th week. This assumption was not confirmed in our research. According to Latour et al. [39], the phenomenon wherein the weight of the yolk sac increases with an increasing amount of yolk is ambiguous. The study showed that the yolk sac weight was the lowest in the group of eggs from hens at 51 weeks old compared to the eggs from 36- and 64-week-old females. In turn, O’Dea et al. [40] and Ulmer-Franco et al. [41] confirmed the lack of correlation between the laying period of the hen and the weight of the yolk sac.

During incubation, changes in the structure of the eggshell occur mainly in places where the network of CAM vessels is developed. The structure does not change at the blunt end of the eggshell [42]. Some authors confirmed that the layers’ age affects the eggshell’s physical properties [43,44,45]. Rodriguez-Navarro et al. [46] found that the female’s age influences the eggshell’s crystallographic texture mainly through different directions of crystallization. Other authors argue that the age of laying hens affects the change in the components in the organic matrix [47,48].

In the groups of 1, 2, and 3-year-old geese, the decrease in eggshell thickness was recorded between the 16th and 22nd day of incubation. Differences in hens’ eggshell thickness occurred at a similar stage of embryonic development (from the 13th day of incubation) [43]. Ye et al. [49] showed a reduction in the eggshell thickness on the 16th day of hatching. Slight changes in the trait were also noted on days 4, 8, and 12. In our research, it was reported that the process of reducing the eggshell thickness was delayed with each subsequent laying period. Similar results were obtained by El-Hanoun et al. [50]. It was found that in a group of younger ducks (from 25 to 35 weeks), the thickness decreased compared to older layers between the ages of 56 and 65 weeks. In duck eggs, it was found that the eggshell thickness was reduced (by 9%) during incubation [51]. In hen eggs, a lower reduction in the eggshell thickness during incubation was noted, namely, a reduction of 6.6% [49].

The developing embryos use the organic component of the eggshell. Mainly, it is the palisade mineral fraction, characterized by a high density in the hatching eggshells [52]. This suggests a relation to the increase in eggshell density. In older layers, changes in eggshell properties may be due to changes in the organic matrix [46].

Water loss from the egg during embryogenesis is a crucial indicator of optimal embryonic development. It is also an indicator of gas exchange. It is correlated with the rate of embryonic metabolism [53]. It was found that the eggshell’s thickness significantly affected the process of water evaporation from the eggs. Water loss from eggs is inversely proportional to eggshell thickness [54,55]. Our research has shown that the age of the female does not affect the total water loss during the hatching of goslings. According to Tona et al. [56], there is a relationship between the female’s age and the egg’s degree of water loss. The eggs’ susceptibility to water loss increases with the age of the layers. This is mainly due to an increase in the conductivity and in the number of pores in the eggshell.

The lowest degree of water loss was characterized in the eggs obtained during incubation at the beginning of laying. Water losses increased with the following weeks of laying. This phenomenon is confirmed by El-Hanoun et al. [50]. By examining the relationship between the females’ age and water loss in duck eggs during incubation, it was shown that with subsequent weeks of laying, the degree of water loss during incubation increases. Iqbal et al. [57] found that hens’ eggs lose less water with successive weeks of laying. Stępińska et al. [58] showed that the degree of water loss in eggs during hatching decreases with the age of turkeys. Based on an egg weight control during storage, less water was lost in the 2nd week of laying compared to the 21^st^ week of laying. A reduced degree of water loss in eggs from young birds during storage may explain higher water losses during incubation. The differences between these published results may be due to the different thermal and humidity conditions during storage and incubation and the hatching technique or type of incubators used. 

The embryo’s temperature depends on the air temperature in the incubator, the amount of heat produced by the embryo, and the heat transfer capacity between the embryo and incubatory environment [59]. It has been shown that the eggshell temperature (EST) during incubation indirectly depends on metabolic changes [60]. The degree of heat production may be related to the size of the egg. It is related to the age of the laying hens. Heat production is higher in older birds than in younger birds. A positive correlation with the size of the yolk has also been considered [56,61]. Comparing the EST in eggs from hens at 30 and 60 weeks of age, Gualhanone et al. [43] showed a lack of correlation between the age of the female and the EST during the hatching. Our research found an influence of age and the laying period on the EST during incubation.

Similarly, Nowaczewski et al. [62] measured the temperature of eggs from hens between 26 and 64 weeks of age. The highest EST was found in the eggshells of hens between 36 and 40 weeks. The EST was similar in all groups during the first 2 days. In the following days, there was a decrease in the EST. This phenomenon corresponds to the loss of moisture by the eggs [59]. Evaporation is a process that consumes a lot of energy. Our research showed an increase in the EST on the 12th day of incubation. Lourens et al. [61] found that heat production increases after the CAM assumes control over breathing functions. 

The allantoic membrane of goose embryos is closed between the 14th and 16th day. Then, metabolic processes intensify, increasing the embryo’s heat production [63]. Our research found that up to the 12th day of incubation, a higher EST characterized the eggs from younger geese compared to the older ones. In the second half of the incubation period, the situation was the opposite: the EST of the eggs from older geese was significantly higher than in other age groups. It can be concluded that the increase in the EST from the group of older females is associated with a higher proportion of CAM in the egg. The incubatory temperature of goose eggs should be 37.8 °C [63]. Our research found that the EST temperature was lower than the incubator air temperature across all the age groups of the females. In hen eggs, at a constant incubation temperature (37.8 °C), the increase in the EST above air temperature occurs from the 10th day of embryogenesis [43,64]. The EST value in the first week is lower than the air temperature in the incubator, probably because the blood vessel network in the chorioallantoic membrane is not fully formed.

## 5. Conclusions

The different embryonic proportions and fetal membranes in the egg and the size of the internal organs indicate a different growth rate and development of the embryos depending on the females’ age and the laying period. The geese’s age influenced the differentiation of thyroxine and triiodothyronine concentrations in the embryos’ blood on day 28, confirming the relationship between the females’ age and the hatching quality. Changes in the eggshell thickness and density during the incubation differed with the geese’s age and the laying period. The decreasing eggshell thickness up to the third season of reproduction was found after the 16th day of incubation, while the reduction in the thickness of the eggs of 4-year-old geese occurred much earlier. A proportional density increase was shown along with a decrease in eggshell thickness for the geese until the third season. In the eggs of the oldest geese, the density was similar throughout the incubatory period. The eggs from the oldest layers were characterized by a lower temperature on the eggshell surface up to the 16th day of hatching compared to the eggs of lower weight and surface area from the younger layers. It is suggested that the chorioallantoic membrane’s formation is associated with an increased eggshell temperature during the hatching period.

## Figures and Tables

**Table 1:** Embryonic development of goose embryos on the 16th day of incubation.

Groups ^1^ n = 15/per Group	Egg Weight (g)	Embryo Weight (g)	Embryo Length (cm)	Share in Egg (%)		Share in the Embryo (%)	
				Embryo	CAM ^2^	Heart	Liver
Female age							
I	153.8 ± 5.9 ^d^	8.81 ± 0.84 ^c^	8.71 ± 0.61 ^c^	5.74 ± 0.61 ^a^	3.84 ± 0.59 ^b^	0.70 ± 0.18 ^b^	1.43 ± 0.24 ^c^
II	176.3 ± 7.6 ^c^	9.57 ± 0.69 ^b^	9.29 ± 0.96 ^ab^	5.46 ± 0.54 ^b^	3.57 ± 0.71 ^b^	0.82 ± 0.16 ^a^	1.98 ± 0.44 ^a^
III	194.4 ± 9.1 ^b^	9.85 ± 0.91 ^ab^	9.40 ± 0.70 ^a^	5.08 ± 0.51 ^c^	4.28 ± 1.06 ^a^	0.74 ± 0.15 ^b^	1.70 ± 0.27 ^b^
IV	211.4 ± 7.8 ^a^	9.99 ± 0.80 ^a^	9.08 ± 0.93 ^b^	4.73 ± 0.46 ^d^	4.40 ± 0.91 ^a^	0.70 ± 0.16 ^b^	1.62 ± 0.29 ^b^
*p*-value	<0.001	<0.001	<0.001	<0.001	0.001	0.028	<0.001
Laying period							
The beginning	188.6 ± 23.4 ^a^	9.43 ± 0.96	8.80 ± 0.65 ^c^	5.04 ± 0.57 ^b^	4.11 ± 1.04 ^a^	0.71 ± 0.18 ^b^	1.72 ± 0.45 ^a^
The peak	179.2 ± 21.5 ^b^	9.64 ± 0.97	9.02 ± 0.53 ^b^	5.43 ± 0.61 ^a^	4.06 ± 0.70 ^a^	0.80 ± 0.16 ^a^	1.71 ± 0.28 ^a^
The end	175.1 ± 21.7 ^c^	9.69 ± 0.92	10.10 ± 0.62 ^a^	5.59 ± 0.74 ^a^	3.66 ± 0.53 ^b^	0.74 ± 0.11 ^ab^	1.54 ± 0.20 ^b^
*p*-value	<0.001	0.255	<0.001	<0.001	0.002	0.001	0.001
Interaction: female age × laying period							
*p*-value	0.003	0.114	<0.001	0.195	<0.001	<0.001	<0.001

^a,b…^, mean values marked with different letters in the columns differ significantly (*p* < 0.05); ^1^—I, 1-year-old geese; II, 2-year-old geese; III, 3-year-old geese; IV, 4-year-old geese; ^2^ CAM, chorioallantoic membrane.

**Table 2:** Embryonic development of goose embryos on the 22nd day of incubation.

Groups ^1^ n = 15/per Group	Egg Weight (g)	Embryo Weight (g)	Embryo Length (cm)	Share in Egg (%)			Share in the Embryo (%)	
				Embryo	Yolk Sac	CAM ^2^	Heart	Liver
Female age								
I	153.3 ± 6.4 ^d^	51.52 ± 4.28 ^c^	17.32 ± 0.63 ^c^	33.61 ± 2.51 ^a^	28.10 ± 3.26 ^b^	2.54 ± 0.47	0.51 ± 0.08	1.66 ± 0.36 ^b^
II	172.6 ± 8.9 ^c^	56.25 ± 5.07 ^b^	18.19 ± 1.03 ^b^	32.60 ± 2.44 ^a^	31.18 ± 3.05 ^a^	2.62 ± 1.15	0.54 ± 0.09	1.82 ± 0.22 ^a^
III	192.5 ± 8.7 ^b^	59.31 ± 4.48 ^a^	18.47 ± 1.07 ^a^	30.82 ± 1.97 ^b^	28.81 ± 3.32 ^b^	2.62 ± 0.41	0.54 ± 0.08	1.79 ± 0.21 ^ab^
IV	210.0 ± 8.1 ^a^	58.67 ± 4.33 ^a^	18.11 ± 0.98 ^b^	27.95 ± 2.02 ^c^	30.35 ± 2.54 ^a^	2.55 ± 0.48	0.52 ± 0.07	1.77 ± 0.24 ^ab^
*p*-value	<0.001	<0.001	<0.001	<0.001	<0.001	0.228	0.247	0.043
Laying period								
The beginning	189.8 ± 22.1 ^a^	59.42 ± 4.57 ^a^	17.30 ± 0.59 ^c^	31.57 ± 3.00	29.19 ± 3.24 ^b^	2.94 ± 0.33 ^a^	0.51 ± 0.06 ^b^	1.76 ± 0.23
The peak	178.9 ± 21.0 ^b^	55.24 ± 4.61 ^b^	18.24 ± 0.80 ^b^	31.11 ± 3.08	30.74 ± 3.71 ^a^	2.38 ± 0.58 ^b^	0.59 ± 0.09 ^a^	1.79 ± 0.19
The end	174.7 ± 21.9 ^c^	53.26 ± 5.24 ^b^	18.95 ± 0.95 ^a^	30.85 ± 3.19	29.04 ± 2.52 ^b^	2.33 ± 0.27 ^b^	0.52 ± 0.06 ^b^	1.74 ± 0.37
*p*-value	<0.001	<0.001	<0.001	0.237	0.009	<0.001	<0.001	0.588
Interaction: female age × laying period								
*p*-value	0.283	0.424	0.001	0.053	0.021	0.984	0.168	0.159

^a,b…^, mean values marked with different letters in the columns differ significantly (*p* < 0.05); ^1^—I, 1-year-old geese; II, 2-year-old geese; III, 3-year-old geese; IV, 4-year-old geese; ^2^ CAM, chorioallantoic membrane.

**Table 3:** Embryonic development of goose embryos on the 28th day of incubation.

Groups ^1^ n = 15/per Group	Egg Weight (g)	Embryo Weight (g)	Embryo Length (cm)	Share in Egg (%)			Share in the Embryo (%)	
				Embryo	Yolk Sac	CAM ^2^	Heart	Liver
Female age								
I	152.3 ± 6.6 ^d^	82.87 ± 9.84 ^d^	22.16 ± 0.84 ^b^	54.33 ± 5.85 ^a^	12.95 ± 2.85 ^d^	1.56 ± 0.17 ^b^	0.43 ± 0.05 ^b^	2.08 ± 0.74
II	174.5 ± 9.9 ^c^	95.81 ± 8.12 ^c^	23.38 ± 0.86 ^a^	54.93 ± 4.02 ^a^	14.60 ± 3.58 ^c^	1.54 ± 0.20 ^b^	0.50 ± 0.08 ^a^	1.97 ± 0.37
III	191.6 ± 8.9 ^b^	103.22 ± 9.81 ^b^	23.43 ± 0.88 ^a^	53.81 ± 4.07 ^a^	15.59 ± 2.94 ^b^	1.64 ± 0.17 ^b^	0.51 ± 0.06 ^a^	1.88 ± 0.28
IV	209.4 ± 9.7 ^a^	108.73 ± 9.21 ^a^	23.27 ± 1.56 ^a^	51.93 ± 3.91 ^b^	17.08 ± 2.87 ^a^	1.94 ± 0.30 ^a^	0.45 ± 0.08 ^b^	1.97 ± 0.55
*p*-value	<0.001	<0.001	<0.001	0.011	<0.001	<0.001	0.04	0.456
Laying period								
The beginning	189.5 ± 22.5 ^a^	102.69 ± 11.75 ^a^	23.35 ± 1.05 ^a^	54.30 ± 3.01	12.87 ± 8.03 ^b^	1.65 ± 0.21	0.49 ± 0.08 ^b^	1.88 ± 0.45 ^b^
The peak	177.9 ± 21.6 ^b^	94.52 ± 12.29 ^b^	22.54 ± 1.38 ^b^	53.27 ± 4.21	13.74 ± 2.69 ^a^	1.71 ± 1.18	0.47 ± 0.07 ^b^	1.88 ± 0.23 ^b^
The end	172.9 ± 21.7 ^c^	91.89 ± 14.22 ^b^	22.37 ± 6.78 ^b^	53.22 ± 6.71	12.71 ± 2.73 ^b^	1.91 ± 0.92	0.53 ± 0.15 ^a^	2.21 ± 0.69 ^a^
*p*-value	<0.001	<0.001	<0.001	0.444	<0.001	0.591	0.032	0.004
Interaction: female age × laying period								
*p*-value	0.226	0.424	<0.001	0.565	0.843	0.998	0.286	0.353

^a,b…^, mean values marked with different letters in the columns differ significantly (*p* < 0.05); ^1^—I, 1-year-old geese; II, 2-year-old geese; III, 3-year-old geese; IV, 4-year-old geese; ^2^ CAM, chorioallantoic membrane.

**Table 4:** Physical features of goose eggshell during incubation.

Feature n = 15/per Group	Group	Eggshell Thickness (mm)			*p*-Value (days) ^A,B…^	Eggshell Density (g × cm^3^)			*p*-Value (days) ^A,B…^
		Day 16	Day 22	Day 31		Day 16	Day 22	Day 31	
Female age ^1^	I	0.525 ± 0.064 ^A^	0.502 ± 0.065 ^AB^	0.477 ± 0.054 ^B^	<0.001	2.107 ± 0.116 ^B^	2.142 ± 0.212 ^Ab^	2.215 ± 0.143 ^Ab^	<0.001
	II	0.513 ± 0.051 ^AB^	0.489 ± 0.042 ^BC^	0.464 ± 0.043 ^C^	<0.001	2.159 ± 0.135 ^C^	2.282 ± 0.213 ^Bb^	2.301 ± 0.083 ^Aa^	<0.001
	III	0.512 ± 0.045 ^A^	0.491 ± 0.085 ^AB^	0.467 ± 0.041 ^B^	<0.001	2.212 ± 0.154	2.260 ± 0.135 ^a^	2.208 ± 0.108 ^b^	0.502
	IV	0.514 ± 0.054 ^A^	0.498 ± 0.054 ^AB^	0.469 ± 0.066 ^B^	<0.001	2.148 ± 0.182	2.203 ± 0.155 ^ab^	2.220 ±0.129 ^b^	0.511
Laying period	The beginning	0.544 ± 0.051 ^Aa^	0.517 ± 0.088 ^Ba^	0.496 ± 0.040 ^Ca^	<0.001	2.228 ± 0.201	2.217 ± 0.126	2.251 ± 0.095 ^a^	0.059
	The peak	0.505 ± 0.087 ^Ab^	0.479 ± 0.047 ^Bb^	0.464 ± 0.040 ^Bb^	<0.001	2.177 ± 0.163	2.184 ± 0.148	2.188 ± 0.168 ^b^	0.434
	The end	0.482 ± 0.039 ^Ac^	0.475 ± 0.041 ^Ab^	0.441 ± 0.058 ^Bc^	<0.001	2.119 ± 0.111 ^B^	2.149 ± 0.225 ^AB^	2.275 ± 0.062 ^Aa^	<0.001
*p*-value ^a,b…^	Female age	0.108	0.955	0.560		0.751	0.005	<0.001	
	Laying period	<0.001	0.001	<0.001		0.286	0.099	<0.001	
	Interaction	0.237	0.217	0.134		0.844	0.054	<0.001	

^a,b…^, the mean values marked with different letters in the columns (female age; laying period) differ significantly (*p* < 0.05); ^A,B…^, the mean values marked with different letters in the rows (days of incubation) differ significantly (*p* < 0.05); ^1^—I, 1-year-old geese; II, 2-year-old geese; III, 3-year-old geese; IV, 4-year-old geese.

**Table 5:** Egg weight loss during incubation (%).

Groups ^1^ n = 15/per Group	Egg Weight (g)	Day of Incubation												
		2	4	6	8	10	12	14	16	18	20	22	24	26
Female age														
I	153.3 ± 6.3 ^d^	1.56 ± 0.73	2.33 ± 0.51 ^b^	3.23 ± 0.65	4.14 ± 0.95	4.97 ± 0.95	5.76 ± 1.08	6.71 ± 1.37	7.58 ± 1.44	8.39 ± 1.67	9.37 ± 1.81	10.43 ± 2.28	11.69 ± 2.20	12.79 ± 2.47
II	173.3 ± 8.84 ^c^	1.61 ± 0.49	2.44 ± 0.63 ^a^	3.42 ± 0.77	4.28 ± 0.91	5.21 ± 1.12	6.02 ± 1.26	7.08 ± 1.96	7.60 ± 1.64	8.66 ± 2.19	9.77 ± 2.04	10.88 ± 2.40	11.92 ± 2.57	13.03 ± 2.86
III	192.56 ± 8.26 ^b^	1.67 ± 0.59	2.65 ± 0.84^a^	3.41 ± 0.71	4.27 ± 0.86	5.11 ± 1.30	5.99 ± 1.26	6.86 ± 1.34	7.67 ± 1.62	8.58 ± 1.79	9.42 ± 2.07	10.56 ± 2.14	11.60 ± 2.39	12.64 ± 2.64
IV	211.07 ± 9.36 ^a^	1.54 ± 0.58	2.42 ± 0.60 ^a^	3.26 ± 0.77	4.08 ± 0.82	4.94 ± 1.00	5.59 ± 1.02	6.83 ± 1.65	7.59 ± 1.42	8.48 ± 1.55	9.38 ± 1.74	10.42 ± 1.93	11.52 ± 2.20	12.60 ± 2.41
*p*-value	<0.001	0.236	<0.001	0.052	0.188	0.103	0.068	0.278	0.244	0.516	0.221	0.251	0.500	0.505
Laying period														
The beginning	189.3 ± 22.3 ^a^	1.66 ± 0.67 ^b^	2.36 ± 0.48 ^b^	3.17 ± 0.57 ^b^	3.99 ± 0.66 ^b^	4.71 ± 0.96^c^	5.42 ± 1.57 ^b^	6.33 ± 0.99 ^b^	7.11 ± 1.08 ^b^	7.89 ± 1.19 ^c^	8.68 ± 1.30 ^b^	9.73 ± 1.72^a^	10.75 ± 1.65 ^b^	11.52 ± 1.76 ^b^
The peak	178.0 ± 21.9 ^b^	1.50 ± 0.43 ^b^	2.31 ± 0.53 ^b^	3.29 ± 0.71 ^b^	4.19 ± 0.98 ^b^	5.11 ± 0.98 ^b^	5.98 ± 1.18 ^b^	7.19 ± 1.97 ^a^	7.92 ± 1.56 ^a^	8.69 ± 1.99 ^b^	9.86 ± 1.86 ^a^	10.89 ± 2.18 ^b^	12.27 ± 2.41 ^a^	13.61 ± 2.54 ^a^
The end	174.3 ± 21.02 ^c^	1.76 ± 0.62 ^a^	2.78 ± 0.94 ^a^	3.65 ± 0.87 ^a^	4.54 ± 1.14 ^a^	5.57 ± 1.28 ^a^	6.35 ± 1.42 ^a^	7.30 ± 1.60 ^a^	8.29 ± 1.79 ^a^	9.35 ± 2.02 ^a^	10.28 ± 2.34 ^a^	11.50 ± 2.44 ^b^	12.38 ± 2.69 ^a^	13.56 ± 3.02 ^a^
*p*-value	<0.001	<0.001	<0.001	<0.001	<0.001	<0.001	<0.001	<0.001	<0.001	<0.001	<0.001	<0.001	<0.001	<0.001
Interaction: female age × laying period														
*p*-value	<0.001	0.337	<0.001	0.348	0.569	0.207	0.593	0.221	0.124	0.316	0.406	0.409	0.421	0.221

^a,b…^, the mean values marked with different letters in the columns differ significantly (*p* < 0.05); ^1^—I, 1-year-old geese; II, 2-year-old geese; III, 3-year-old geese; IV, 4-year-old geese.

## Data Availability

This research was part of the first author’s doctoral dissertation. Thesis: Joanna Kucharska-Gaca. 2018. The analysis of embryonic and post-embryonic development of geese depends on the age of the parent flock. Publisher: UTP–University of Science and Technology in Bydgoszcz. Repozytorium Cyfrowe Politechniki Bydgoskiej–Analiza rozwoju embrionalnego i postembrionalnego gęsi w zależności od wieku stada rodzicielskiego (utp.edu.pl) (doctoral thesis).

## References

[1] Sechman A., Bobek S. (1997). Concentration of thyroid hormones in the yolk of avarian follicles during the ovulatory cycle of hen. [Stężenie hormonów tarczycy w żółtku pęcherzyków jajnikowych kury w czasie cyklu owulacyjnego]. Zesz. Nauk. Przeg. Hod..

[2] Christensen V.L., Donaldson W.E., McMurtry J.P. (1996). Physiological differences in late embryos from turkey breeders at different ages. Poult. Sci..

[3] Czarnecki C.M. (1991). Influence of exogenous T4 on body weight, feed consumption, T4 levels, and myocardial glycogen in furazolidone-fed turkey poults. Avian Dis..

[4] Króliczewska B. (2014). Selected hormones in embryonic development and bird growth. [Wybrane hormony w rozwoju embrionalnym i wzroście ptaków]. Pol. Drob..

[5] Gardahaut M.F., Fontaine-Perus J., Rouaud T., Bandman E., Ferrand R. (1992). Developmental modulation of myosin expression by thyroid hormone in avian skeletal muscle. Development.

[6] Hughes S.M., Taylor J.M., Tapscott S.J., Gurley C.M., Carter W.J., Peterson C.A. (1993). Selective accumulation of MyoD and Myogenin mRNAs in fast and slow adult skeletal muscle is controlled by innervation and hormones. Development.

[7] Stockdale F.E. (1992). Myogenic cell lineages. Dev. Biol..

[8] Kim J.W. (2010). The endocrine regulation of chicken growth. Asian-Australas. J. Anim. Sci..

[9] King D.B., King C.R. (1976). Thyroidal influence on early muscle growth of chickens. Gen. Comp. Endocrinol..

[10] Christensen V.L., Donaldson W.E., Nestor K.E. (1993). Embryonic viability and metabolism in turkey lines selected for egg production or growth. Poult. Sci..

[11] Christensen V.L., Wineland M.J., Fasenko G.M., Donaldson W.E. (2001). Egg storage effects on plasma glucose and supply and demand tissue glycogen concentrations of broiler embryos. Poult. Sci..

[12] Morris T.R. (2004). Environmental control for layers. World Poult. Sci. J..

[13] Olanrewaju H.A., Thaxton J.P., Dozier W.A., Purswell J.L., Roush W.B., Branton S.L. (2006). A review of lighting programs for broiler production. Int. J. Poult. Sci..

[14] Buyse J., Kuhn E.R., Decuypere E. (1996). The use of intermittent lighting in broiler raising. 1. Effect on broiler performance and efficiency of nitrogen retention. Poult. Sci..

[15] Manser C.E. (1996). Effects of lighting on the welfare of domestic poultry: A review. Anim. Welf..

[16] Powell D.J., Velleman S.G., Cowieson A.J., Singh M., Muir W.I. (2016). Influence of chick hatch time and access to feed on broiler muscle development. Poult. Sci..

[17] Hulet R., Gladys G., Hill D., Meijerhof R., El-Shiekh T. (2007). Influence of Egg Shell Embryonic Incubation Temperature and Broiler Breeder Flock Age on Posthatch Growth Performance and Carcass Characteristics. Poult. Sci..

[18] Kucharska-Gaca J., Adamski M., Biesek J. (2022). The age of the geese from the parent flock and the laying period affect the features of the eggs. Poult. Sci..

[19] Rząsa J., Sechman A., Bednarczyk M., Mika M., Rosiński A., Paczoska-Eliasiewicz H. (2000). Pattern of plasma thyroid and gonadal hormones concentrations during embryogenesis in two strains of white Italian goose. Brit. Poult. Sci..

[20] Reyns G.E., Venken K., Morreale de Escobar G., Kuhn E.R., Darras V.M. (2003). Dynamics and regulation of intercellular thyroid hormone concentrations in embryonic chicken liver, kidney, brain and blood. Gen. Comp. Endocr..

[21] Lu J.W., McMurtry J.P., Coon C.N. (2007). Developmental changes of plasma insulin, glucagon, insulin-like growth factors, thyroid hormones and glucose concentrations in chick embryos and hatched chicks. Poult. Sci..

[22] De Groef B., Grommen S.V.H., Darras V.M. (2013). Hatching the cleidoic egg: The role of thyroid hormones. Front. Endocrinol..

[23] Yahav S., Collin A., Shinder D., Picard M. (2004). Thermal manipulations during broiler chick embryogenesis: Effects of timing and temperature. Poult. Sci..

[24] Pietras M., Herbut E., Barowicz T. (1996). Effect of thyrotropin releasing hormone on the level of thyroid hormones, oxygen consumption and growth of broiler cockerels reared under various thermal conditions. [Wpływ hormonu uwalniającego tyreotropinę na poziom hormonów tarczycy, zużycie tlenu i wzrost kogutów brojlerów odchowywanych w różnych warunkach termicznych]. Rocz. Nauk. Zoot..

[25] Tong Q.C., Romanini E., Exadaktylos V., Bahr C., Berckmans D., Bergoug H., Eterradossi N., Roulston N., Verhelst R., McGonnell I.M. (2013). Embryonic development and the physiological factors that coordinate hatching in domestic chickens. Poult. Sci..

[26] Wilson H.R. (1997). Interrelationships of egg size, chick size, post-hatching growth and hatchability. World Poult. Sci. J..

[27] Nangsuay A., Ruangpanit Y., Meijerhof R., Attamangkune S. (2011). Yolk absorption and embryo development of small and large eggs originating from young and old breeder hens. Poult. Sci..

[28] Polonis A., Dmoch M. (2007). Role and significance of yolk sac in the pre- and posthatching periods in chickens. [Rola i znaczenie woreczka żółtkowego w okresie pre- i postnatalnym piskląt]. Życie Wet..

[29] Şahan U., Ipek A., Sozcu A. (2014). Yolk sac fatty acid composition, yolk absorption, embryo development, and chick quality during incubation in eggs from young and old broiler breeders. Poult. Sci..

[30] Yadgary L., Cahaner A., Kedar O., Uni Z. (2010). Yolk sac nutrient composition and fat uptake in late-term embryos in eggs from young and old broiler breeder hens. Poult. Sci..

[31] Morita V.S., Boleli I.C., Cargnelutti Filho A. (2009). Hematological values and body, heart and liver weights of male and female broiler embryos of young and old breeder eggs. Rev. Bras. Cienc. Avic..

[32] Hudson B.P., Fairchild B.D., Wilson J.L., Dozier W.A., Buhr R.J. (2004). Breeder age and zinc source in broiler breeder hen diets on progeny characteristics at hatching. J. Appl. Poult. Res..

[33] Zhang H., Burggren W.W. (2012). Hypoxic level and duration differentially affect embryonic organ system development of the chicken (*Gallus gallus*). Poult. Sci..

[34] Mróz E., Łepek G. (1999). Control of allantoic development in goose eggs. [Kontrola rozwoju omoczni w jajach gęsich]. Zesz. Nauk. Przeg. Hod..

[35] Mortola J.P., Awam K.A. (2010). Growth of the chicken embryo: Implications of egg size. Comp. Biochem. Physiol. A Mol. Integr. Physiol..

[36] Tullett S.G., Deeming D.C. (1987). Failure to turn eggs during incubation: Effects on embryo weight, development of the chorioallantois and absorption of albumen. Brit. Poult. Sci..

[37] Lewis P.D., Perry G.C., Koutoulis K.C. (1998). Correlations between yolk size and age, egg weight or body weight at sexual maturity. Brit. Poult. Sci..

[38] Reidy T.R., Atkinson J.L., Leeons S. (1998). Size and components of poultry yolk sac. Poult. Sci..

[39] Latour M.A., Peebles E.D., Boyle C.R., Doyle S.M., Pansky T., Brake J.D. (1996). Effects of breeder hen age and dietary fat on embryonic and neonatal broiler serum lipids and glucose. Poult. Sci..

[40] O’Dea E.E., Fasenko G.M., Feddes J.J.R., Robinson F.E., Segura J.C., Ouellette C.A., Van Middelkoop J.H. (2004). Investigating the eggshell conductance and embryonic metabolism of modern and unselected domestic avian genetic strains at two flock ages. Poult. Sci..

[41] Ulmer-Franco A.M., Fasenko G.M., O’Dea E.E. (2010). Hatching egg characteristics, chick quality, and broiler performance at 2 breeder flock ages and from 3 egg weights. Poult. Sci..

[42] Doskocil M., Blazek J., Nĕmcová P., Stárková B. (1985). Structure of the hen’s egg shell and its changes during incubation. A scanning electron microscope study. Anat. Anz..

[43] Gualhanone A., Furlan R.L., Fernandez-Alarcon M.F., Macari M. (2012). Effect of breeder age on eggshell thickness, surface temperature, hatchability and chick weight. Braz. J. Poult. Sci..

[44] Pakulska E., Badowski J., Bielińska H., Bednarczyk M. (2003). Influence of age on physical characteristics of eggs and hatchability of white Kołuda goslings. [Wpływ wieku na cechy fizyczne jaj i wylęgowość piskląt gęsi Białych Kołudzkich]. Zesz. Nauk. Przeg. Hod..

[45] Połtowicz K., Calik J. (2002). The influence of age and origin of laying hens on the quality of food egg shells. [Wpływ wieku i pochodzenia kur nieśnych na jakość skorup jaj spożywczych]. Rocz. Nauk. Zoot..

[46] Rodriguez-Navarro A., Kalin O., Nys Y., Garcia-Ruiz J.M. (2002). Influence of the microstructure on the shell strength of eggs laid by hens of different ages. Brit. Poult. Sci..

[47] Fraser A.C., Bain M.M., Solomon S.E. (1998). Organic matrix morphology and distribution in the palisade layer of eggshells sampled at selected periods during lay. Brit. Poult. Sci..

[48] Panheleux M., Nys Y., Williams J., Gautron J., Boldicke T., Hincke M.T. (2000). Extraction and quantification by ELISA of eggshell organic matrix proteins (ovocleidin-17, ovalbumin, ovotransferrin) in shell from young and old hens. Poult. Sci..

[49] Yue Y., Zhanming L., Jinming P. (2016). Changes in pigment, spectral transmission and element content of pink chicken eggshells with different pigment intensity during incubation. PeerJ.

[50] El-Hanoun A.M., Rizk R.E., Shahein E.H.A., Hassan N.S., Brake J. (2012). Effect of incubation humidity and flock age on hatchability traits and posthatch growth in Pekin ducks. Poult. Sci..

[51] Balkan M., Karakas R., Biricik M. (2006). Changes in eggshell thickness, shell conductance and pore density during incubation in the Peking duck (*Anas platyrhynchos* f. dom.). Ornis Fenn..

[52] Ketta M., Tůmová E. (2016). Eggshell structure, measurements, and quality-affecting factors in laying hens: A review. Anim. Sci..

[53] Araújo I.C.S.I., Leandro N.S.M.I., Mesquita M.A.I., Café M.B.I., Mello H.H.C.I., Gonzales E.I. (2016). Effect of incubator type and broiler breeder age on hatchability and chick quality. Braz. J. Poult. Sci..

[54] Tullett S.G., Board R.G. (1977). Determinants of avian eggshell porosity. J. Zool..

[55] Joseph N.S., Moran E.T. (2005). Effect of flock age and postemergent holding in the hatcher on broiler live performance and further processing yield. J. Appl. Poult. Res..

[56] Tona K., Bamelis F., Coucke W., Bruggeman V., Decuypere E. (2001). Relationship between broiler breeder’s age and egg weight loss and embryonic mortality during incubation in largescale conditions. J. Appl. Poult. Res..

[57] Iqbal J., Khan S.H., Mukhtara N., Tanveer A., Riaz A.P. (2016). Effects of egg size (weight) and age on hatching performance and chick quality of broiler breeder. J. Appl. Anim. Res..

[58] Stępińska M., Mróz E., Krawczyk M., Otowski K., Górska A. (2016). Effect of hen age and storage time on egg weight loss and hatchability results in turkeys. Ann. Anim. Sci..

[59] Meijerhof R. (2001). Embryo temperature—A new parameter in the hatchery? [Temperatura zarodka–nowy parametr w zakładzie wylęgowym?]. Pol. Drob..

[60] Lourens A., Van den Brand H., Meijerhof R., Kemp B. (2005). Effect of eggshell temperature during incubation on embryo development, hatchability and post-hatch development. Poult. Sci..

[61] Lourens A., Molenaar R., Van den Brand H., Heetkamp M.J., Meijerhof W.R., Kemp B. (2006). Effect of egg size on heat production and the transition of energy from egg to hatchling. Poult. Sci..

[62] Nowaczewski S., Babuszkiewicz M., Kaczmarek S. (2016). Effect of broiler breeders age on eggshell temperature, embryo viability and hatchability parameters. Ann. Anim. Sci..

[63] Badowski J. (1998). Review of issues related to the hatching technology of goose eggs. [Przegląd problematyki związanej z technologią lęgu jaj gęsich]. Biul. Inf. IZ..

[64] Lis M.W. (2012). Bird embryo thermoregulation part I. Measurements of egg shell temperature. [Termoregulacja zarodka ptaków cz. I. Pomiary temperatury skorupy jaj]. Pol. Drob..

